# Malaria and COVID-19 prevalence in a population of febrile children and adolescents living in Libreville

**DOI:** 10.4102/sajid.v37i1.459

**Published:** 2022-10-26

**Authors:** Bridy C. Moutombi Ditombi, Bedrich Pongui Ngondza, Charleine Manomba Boulingui, Ornella A. Mbang Nguema, Jack M. Ndong Ngomo, Noe P. M’Bondoukwé, Reinne Moutongo, Denise P. Mawili-Mboumba, Marielle K. Bouyou Akotet

**Affiliations:** 1 Department of Parasitology-Mycology and Tropical Medicine, Faculty of Medicine, Université des Sciences de la Santé, Libreville, Gabon; 2 Operational Research Clinical Unit, Hôpital Régional Estuaire Melen, Libreville, Gabon; 3 Department of Medicine, Faculty of Medicine, Université des Sciences de la Santé, Libreville, Gabon; 4 Unité Mixte de Recherche en Agents Infectieux et Pathoogies Assosciées, UMRAIP, Université des Sciences de la Santé, Owendo, Gabon

**Keywords:** malaria, COVID-19, co-infection, Gabon, children, adolescents

## Abstract

**Background:**

Patients with acute febrile illness need to be screened for malaria and coronavirus disease 2019 (COVID-19) in malaria-endemic areas to reduce malaria mortality rates and to prevent the transmission of the severe acute respiratory syndrome coronavirus 2 (SARS-CoV-2).

**Objectives:**

To estimate the frequency of children and adolescents with COVID-19 and/or malaria among febrile patients attending for malaria diagnosis

**Method:**

This cross-sectional study was conducted in a sentinel site for malaria surveillance during the SARS-CoV-2 pandemic (Omicron variant), from October 2021 to December 2021 in Gabon. All febrile patients were tested for malaria using microscopy. Severe acute respiratory syndrome coronavirus 2 was detected by real time polymerase chain reaction (RT-PCR) and rapid antigen tests developed by Sansure Biotech^®^.

**Results:**

A total of 135 patients were screened. Their median age was 6 (interquartile range [IQR]: 3–14) years. Malaria was confirmed for 49 (36.3%) patients, 29 (32.5%) children, 13 (59.0%) adolescents and 7 (29.2%) adults. The frequency of COVID-19 cases was 7.4% (*n* = 10/135), and it was comparable between children (*n* = 6; 6.7%), adolescents (*n* = 2; 9.1%) and adults (*n* = 2; 8.3%) (*p =* 0.17). Malaria and COVID-19 co-infections were diagnosed in 3 (6.1%) patients from all the age groups. Participants with a co-infection had a higher median temperature, a higher median parasitaemia, and were mostly infected with non-*falciparum* malaria.

**Conclusion:**

COVID-19 cases and cases of malaria/COVID-19 co-infections were found in febrile children and adolescents. SARS-CoV-2 testing should be included in the screening of suspected malaria cases.

**Contribution:**

This study highlights the presence of malaria-COVID-19 coinfection among children and adolescents who should also be screened for both diseases, like for adults.

## Introduction

Coronavirus disease 2019 (COVID-19), caused by the severe acute respiratory syndrome coronavirus 2 (SARS-CoV-2), emerged in Wuhan, China, in December 2019. It has since been responsible for more than 6 million deaths worldwide.^1^ The COVID-19 global pandemic has placed an additional burden on health care systems, and raised new challenges for the management of other febrile illnesses in co-endemic settings.^2^ In 2020, a total of 241 million cases of malaria and 627 000 deaths were reported worldwide.^3^ In Gabon, 35% of febrile cases are caused by malaria (data from malaria sentinel site). Most febrile illnesses include non-specific symptoms. The clinical symptoms of COVID-19 such as fever, myalgia, asthenia, headaches, shortness of breath and gastrointestinal problems may, however, overlap with the symptoms of uncomplicated or severe malaria.^4^ Asymptomatic COVID-19 patients maintain the SARS-CoV-2 transmission. Thus, suspected cases of malaria should be fully screened for COVID-19, irrespective of the presence of respiratory signs and symptoms, in order to establish the right diagnosis and provide proper treatments. Cases of SARS-CoV-2 with bacterial, viral or plasmodial co-infections have been reported in the literature but data from African settings are scarce.^5,6^ In Gabon, where perennial malaria transmission is either meso-endemic or hyperendemic, febrile individuals attend malaria sentinel sites throughout the country to obtain a diagnosis and seek medical care. Children and teenagers represent more than 70% of these patients. The concomitant screening of malaria and COVID-19 could contribute to reduce malaria-related deaths, to prevent the transmission of COVID-19 from children to vulnerable relatives, and to avoid life-threatening conditions on account of delayed treatment.

The objective of this study was to estimate the number of COVID-19 cases and malaria/COVID-19 co-infection cases among children and adolescents attending a malaria sentinel site during the outbreak of Omicron SARS-CoV-2 in Gabon.

## Methodology

A cross-sectional study was conducted from October 2021 to December 2021 at the malaria sentinel site in the Melen Regional Hospital, Gabon. The main activity of the sentinel site consists in screening febrile patients for malaria during consultations at the hospital. Patients were recruited during the peak of the third wave of COVID-19 (Omicron variant).

All febrile patients or patients with a history of fever, who gave their oral consent, benefitted from Giemsa-stained thick and thin blood smears for microscopic malaria diagnosis. Nasopharyngeal and oropharyngeal swabs were collected and analysed to detect SARS-CoV-2 antigens using rapid diagnostic tests (RDTs) (Sansure Biotech^®^ SARS-CoV-2 Rapid Antigen Test) as per the manufacturer’s instructions, and according to the National COVID-19 Response Committee (COPIL-Gabon) guidelines for rapid isolation. Real time polymerase chain reaction (RT-PCR) was also performed using RT-PCR SARS-CoV-2 Sansure Biotech Novel Coronavirus kits (2019-nCoV).

Age, previous antimalarial treatment, bed net use, temperature, main clinical symptoms, lymphocytes and platelets count were recorded. According to their age, patients were classified as follows: children (below 12 years old), teenagers or adolescents (from 12 to 17 years old) and adults (above 18 years old).

Patients with a positive SARS-CoV-2 test were referred for COVID-19 care at dedicated outpatient treatment centres. Patients with malaria were treated with artemisinin-based combination therapies (ACT) according to local recommendations.

## Statistical analysis

All data were entered twice and recorded in an Excel spreadsheet, continuous data are summarised as median (25e–75e interquartile range), and qualitative data as proportions (%). Differences across groups were analysed using the Chi-squared test, Fisher’s exact test, the Kruskall–Wallis test or Mann–Whitney test. All analyses were performed using Statview 5.0 software.

## Results

A total of 135 patients accepted to be tested for SARS-CoV-2, interviewed and screened. Their median age was 6 (interquartile range [IQR]: 3–14) years and 79.2% (*n* = 107/135) were aged below 15 years, with children being predominant (*n* = 89/135) (Table 1). Almost three quarters of patients (73.3%; *n* = 99/135) lived in an urban area and nearly one-third (32.6%; *n* = 44/135) used insecticide-treated bed nets. Their characteristics are presented in Table 1.

**TABLE 1:** Main characteristics of the study participants.

Variables	*n*	%
**Age (year)**		
Children	89	65.9
Adolescents	22	16.3
Adults	24	17.8
**Gender**		
Female	71	52.6
Male	64	47.4
**Insecticide treated nets (ITN)**	44	32.6
**Self-medication with an antimalarial drug**	41	30.4
**Symptoms**		
Cough	63	46.7
Rhinorrhoea	28	20.7
Influenza symptoms	19	14.1
**Malaria**	49	36.3
**Coronavirus disease 2019**	10	7.4
**Malaria species**		
*Plasmodium malariae*	3	6.1
*Plasmodium falciparum*	46	93.9

The plasmodial infection rate was below 40% and the median parasite density was 445 (130–4270) parasites/µL. *Plasmodium falciparum* was identified in more than 90% of the malaria positive patients, followed by *Plasmodium malariae* (Table 1). Among the 49 patients with plasmodial infection, three patients had a positive COVID-19 test (6.1%, *n* = 3/49). The other patients consulting for fever (*n* = 79/135; 58.5%) had negative malaria and COVID-19 tests.

Children and adolescents were predominant (*n* = 8/10) among those with a COVID-19 infection; indeed, SARS-CoV-2 was detected in 9.1% (*n* = 2/22) of the adolescents, 8.4% (*n* = 2/24) of adults and 6.7% (*n* = 6/89) of children (*p =* 0.17) (Table 2). Patients infected with SARS-CoV-2 tended to be older (12 [IQR: 7–24] years) than those with other febrile illnesses (6 [IQR: 2–14] years) and those with malaria (9 [IQR: 6–17] years) (*p* = 0.08). There was no significant association between single malaria, single COVID-19, co-infection and the gender (Table 2).

**TABLE 2:** Single and co-infection frequency according to age and gender.

Variables	Malaria				Co-infection				COVID-19				*p*
	*n*	%	Median	IQR	*n*	%	Median	IQR	*n*	%	Median	IQR	
**Age**	-	-	7	3–14	-	-	14	5–20	-	-	10	7–14	0.42
Children	28	31.4	-	-	1	1.1	-	-	5	5.6	-	-	0.07
Adolescents	12	54.5	-	-	1	4.5	-	-	1	4.5	-	-	-
Adults	6	25.0	-	-	1	4.2	-	-	1	4.2	-	-	-
**Gender**													
Female	20	28.2	-	-	2	2.8	-	-	5	7.0	-	-	0.53
Male	26	40.6	-	-	1	1.6	-	-	2	3.1	-	-	-

COVID-19, coronavirus disease 2019; IQR, interquartile range.

Most participants had taken an antimalarial drug prior to the screening. Although fever duration was comparable between the three groups, most of the children with malaria had symptoms since more than 6 days when parents sought for diagnosis (Table 3). All patients with co-infections still had fever on the day of the consultation; the child and the adolescent temperature was 40 °C, while it was 38 °C for the adult. Co-infected participants also had the highest median parasitaemia (*p* < 0.01) (Table 3). Among the three patients with non-*falciparum* malaria, two had a COVID-19 co-infection. Thrombocytopenia was the predominant biological sign in patients with malaria (Table 3).

**TABLE 3:** Characteristics of single and malaria and COVID-19 co-infection.

Variables	Malaria (*N* = 46)				Co-infection (*N* = 3)				COVID-19 (*N* = 7)				*p*
	*n*	%	Median	IQR	*n*	%	Median	IQR	*n*	%	Median	IQR	
**Antimalarial self-medication**	42	91.3	-	-	3	100.0	-	-	7	100.0	-	-	0.7
**Duration of fever**	-	-	3	2.0–3.0	-	-	3	1.5–4.0	-	-	2	1.2–3.0	0.2
< 3 days	20	43.5	-	-	1	33.3	-	-	5	71.4	-	-	0.3
3–6 days	21	45.6	-	-	2	66.7	-	-	2	28.6	-	-	-
≥ 1 week	5	10.7	-	-	0	0.0	-	-	0	0.0	-	-	-
**Temperature (°C)**	-	-	38	37.0–39.0			40	39.0–40.0		-	37	37.0–38.0	< 0.01
**Presence of fever**	33	71.7	-	-	3	100.0	-	-	2	28.6	-	-	0.03
**Parasitaemia (p†/µL)**	-	-	409	100–4200	-	-	466	254–22 017	-	-	-	-	< 0.01
Parasite species													
*Plasmodium falciparum*	44	95.7	-	-	1	33.3	-	-	-	-	-	-	-
*Plasmodium malariae*	2	4.3	-	-	2	66.7	-	-	-		-	-	-
**Lymphocytes, 10^3^/µL (*n* = 38)**	-	-	1.8	1.0–2.4			1.2	1.1–3.2	-	-	2.5	1.6–3.0	0.33
Lymphopenia	6	15.8	-	-	-	-	-	-	1	14.2	-	-	-
Lymphocytosis	7	18.4	-	-	-	-	-	-	0	0.0	-	-	0.47
**Platelets, 10^3^/µL (*n* = 38)**	-	-	146	84–235	-	-	139	52–185	-	-	220	199–283	< 0.01
Thrombopenia	18	47.4	-	-	2	66.7	-	-	0	0.0	-	-	0.01

COVID-19, coronavirus disease 2019.

† , p = parasites.

## Discussion

In malaria sentinel sites, patients with fever or a history of fever are routinely screened for malaria symptoms that overlap those presented by individuals infected with SARS-CoV-2. Making a diagnosis based on the aetiology of these diseases has now become more difficult for patients attending health structures in malaria endemic settings. The use of malaria RDT is now the rule for the rapid screening of malaria for suspected cases. All patients with a positive PCR also had a positive antigen test, indicating that Sansure Biotech^®^ RDT are reliable COVID-19 antigen tests which can be introduced for patient triage and rapid isolation at health care entry points.

The present results highlight the non-negligible number of COVID-19 cases in youths exhibiting symptoms of malaria (7.4%) compared to the national rates of COVID-19 prevalent at the time of the study (4.7%).^7^ Febrile adolescents seemed to have the same risk of being infected by the SARS-CoV-2 as for the adults; the risk of infection also exists among young children.

Cases of malaria/COVID-19 co-infection have been previously studied in populations of COVID-19 patients. The prevalence of co-infections ranges between 1% and 40% in the literature, with an average of 11%.^8,9^ *Plasmodium*/SARS-CoV-2 co-infections were also observed in COVID-19 patients hospitalised in Libreville, the capital city of Gabon. The rate of these co-infections was found within the range of that observed elsewhere (3%).^10,11^

However, investigations performed in children and teenagers attending malaria health facilities are scarce. This observational study emphasises the need to screen patients of all ages for non-malarial febrile illnesses in health facilities, including COVID-19, as the prevalence of febrile illnesses is high (58.5%). Indeed, all patients presented symptoms that are frequent during mild malaria or COVID-19 infections.

In this study, only 6.1% of patients had a COVID-19/malaria co-infection. This rate is consistent with those described by other studies in co-endemic African settings.^12,13^ Although most patients exhibited mild symptoms, the present study indicates that SARS-CoV-2 infection worsened the fever of patients with malaria which had temperature higher than 39° C. A co-infection could lead to convulsions and other neurological complications in children.^14^ Moreover, the parasitaemia load was also higher in participants with a co-infection, although it remained below the level described in the 12 cases series described in the literature.^15,16^ The low rate of co-infection, the fact that children and teenagers are less prone to severe COVID-19 symptoms, and that none of the screened patients had severe malaria, could also explain this observation. In addition, the species *P. malariae* was detected in two out of the three children with co-infection. The first case series of malaria/COVID-19 co-infections associated either *Plasmodium vivax* or *Plasmodium ovale* with SARS-CoV-2.^17,18,19^ Although no conclusion can be drawn because of the low sample size in our study, the association between non-*Falciparum* species and SARS-CoV-2 should be investigated in further studies with larger sample groups.

Moreover, the rate of self-medication with antimalarial drugs among COVID-19 participants and patients with non-malarial febrile illnesses can be a point of concern. Community sensitisation and awareness campaigns to recommend the systematic screening for, at the very least, malaria and COVID-19 in the case of fever, as well as the introduction of COVID-19 RDT for the screening of patients with febrile illnesses, are necessary.

In conclusion, COVID-19 infections and COVID-19/malaria co-infections should be screened in febrile children and adolescents in malaria endemic settings and investigated in other settings with a larger sample size.
